# Neonatal Febrile Seizures in Rats Induce Long-Term Region-Specific Alterations in the Glutamatergic System of Hippocampal–Prefrontal Circuits and Lead to Behavioral Deficits

**DOI:** 10.3390/cells14211666

**Published:** 2025-10-23

**Authors:** Alexandra V. Griflyuk, Olga E. Zubareva, Anna A. Kovalenko, Maria V. Zakharova, Aleksey V. Zaitsev

**Affiliations:** Sechenov Institute of Evolutionary Physiology and Biochemistry of RAS, 44, Toreza Prospekt, Saint Petersburg 194223, Russia; griflyuk.al@mail.ru (A.V.G.); zubarevaoe@mail.ru (O.E.Z.); kovalenko_0911@mail.ru (A.A.K.); zaharova-masha@yandex.ru (M.V.Z.)

**Keywords:** febrile seizures, rat model, ventral hippocampus, medial prefrontal cortex, NMDA, glutamatergic system, mGluR, EAAT, anxiety-like behavior

## Abstract

**Highlights:**

- This study provides the first comprehensive analysis of long-term, region-specific changes in the entire glutamatergic system (iGluRs, mGluRs, EAATs) after neonatal febrile seizures.
- The most severe and persistent molecular alterations occur in the ventral hippocampus and medial prefrontal cortex, key nodes for emotional regulation and cognition.
- The behavioral outcome is characterized by a hyperanxious phenotype with locomotor and habituation deficits, but with working memory and sociability remaining unaffected.
- The findings reveal a precise neurobiological mechanism that could underlie the increased risk of anxiety-related disorders following early-life febrile seizures.

**Abstract:**

Febrile seizures (FS) are a common childhood neurological event associated with an increased risk of long-term cognitive and emotional deficits, though the precise mechanisms remain elusive. Using a rat model, we investigated the long-term effects of FS induced on postnatal day 10, assessing outcomes in young adulthood (P45-55). We report region-specific neuronal loss in the hippocampus, more extensive in the ventral segment. Molecular analysis revealed a broad downregulation of genes encoding ionotropic and metabotropic glutamate receptors and excitatory amino acid transporters. These alterations were most severe and persistent in the ventral hippocampus and medial prefrontal cortex. Behaviorally, rats with neonatal FS exhibited a hyperanxious phenotype, characterized by reduced locomotor and exploratory activity and impaired habituation to a novel environment. In contrast, spatial working memory and social behavior remained intact. Our results provide the first comprehensive evidence that neonatal FS trigger long-term, region-specific disruptions of the glutamatergic system within hippocampal–prefrontal circuits. These findings identify vulnerable molecular targets and precise neurobiological mechanisms that may underlie the heightened risk of anxiety-related disorders following early-life FS, suggesting new avenues for therapeutic intervention.

## 1. Introduction

Febrile seizures (FS) are one of the most prevalent neurological disorders in children aged six months to five years, with a peak incidence in the second year of life [1]. These seizures typically occur at body temperatures above 38 °C in the absence of other triggering factors like CNS infection or trauma [1]. Although most FS episodes are benign, complex forms—such as those that are prolonged (>15 min), recurrent within 24 h, or progress to febrile status epilepticus—are associated with an elevated risk of cognitive impairments and mental disorders later in life, including schizophrenia [2], attention deficit hyperactivity disorder (ADHD) [3,4], and autism spectrum disorders [5]. Despite the established link, the specific disorders that arise after FS and which may subsequently cause these disorders remain unclear. Experimental models of FS also replicate cognitive and psychoemotional deficits [6,7,8,9,10,11,12], although the precise molecular mechanisms behind these impairments remain poorly understood.

The early postnatal period represents a critical window for the development of limbic and cortical circuits that support cognitive and emotional functions [13]. We hypothesize that FS during this vulnerable period disrupts the developmental trajectory of these circuits, thereby contributing to long-term impairments.

Clinical and electroencephalographic (EEG) studies implicate the hippocampus in FS in children [14,15] and animal models [16]. This structure, vital for learning, memory, and emotion [17,18], is highly vulnerable to hyperexcitability and a key focus in post-seizure cognitive research. Critically, the hippocampus is not uniform; it exhibits a functional dorsoventral gradient [17]. The dorsal hippocampus in rats is primarily involved in spatial memory, while the ventral part regulates emotional behaviors like anxiety and fear [18,19]. This regional specificity means negative impacts can be locally confined [20], a nuance often overlooked.

However, a singular focus on the hippocampus is insufficient to understand the long-term consequences of FS. Higher-order functions are mediated by distributed networks that include the medial prefrontal cortex (mPFC) and temporal cortex. The mPFC is crucial for executive functions and emotion regulation [21,22,23], and the temporal cortex for semantic memory and sensory processing [24,25]. These areas are densely interconnected with the hippocampus [21,22,26], and their dysfunction can significantly contribute to the cognitive and emotional deficits following FS. Consequently, a comprehensive investigation of impairments across all these interconnected brain regions is necessary to fully understand the long-term consequences of FS.

The pathogenesis of FS and its associated neuropsychiatric sequelae primarily involves genetic predispositions, hyperthermia-mediated mechanisms, and neuroinflammatory pathways [27]. Genetic factors prominently contribute through mutations in ion channel genes, including sodium channel subunits (*SCN1A*, *SCN1B*), GABA_A_ receptor subunits (*GABRG2*, *GABRD*), and hyperpolarization-activated channels (*HCN1*, *HCN2*). These defects disrupt ionic homeostasis and reduce inhibition, predisposing to seizures [27,28].

Fever-induced hyperthermia directly triggers FS by modulating temperature-sensitive ion channels (TRP, TREK-1, HCN), causing membrane depolarization and increased excitability [27,29].

Simultaneously, inflammatory responses are central to FS. Proinflammatory cytokines, particularly IL-1β, enhance NMDA receptor function, impair glutamate clearance, and reduce GABAergic inhibition, promoting hyperexcitability [30,31]. IL-6 and TNF-α further destabilize excitation–inhibition balance [28,32]. The critical role of inflammation is confirmed by elevated seizure thresholds when IL-1 signaling is blocked [30,31].

Crucially, key FS mechanisms—notably neuroinflammation and hyperthermia—converge on the glutamatergic system [20,33]. We propose that this convergence underlies the prolonged neuronal hyperexcitability and behavioral disturbances seen following FS [34,35,36]. The impact of these pathological processes is likely most severe during early postnatal development, a critical period for the maturation of glutamate networks [37]. We therefore conducted a comprehensive investigation of the persistent, FS-induced alterations within the glutamatergic system.

Excessive release of glutamate and subsequent overactivation of its receptors represent the primary mechanism of excitotoxicity during seizures [38,39,40]. This cascade triggers dysregulated calcium influx, oxidative stress, and ultimately, neuronal death. Ionotropic glutamate receptors (iGluRs), specifically N-methyl-D-aspartate (NMDA) and α-amino-3-hydroxy-5-methyl-4-isoxazolepropionic acid (AMPA) receptors, are key players in this process. Furthermore, metabotropic glutamate receptors (mGluRs), which modulate synaptic transmission and plasticity, and excitatory amino acid transporters (EAATs), responsible for glutamate reuptake and homeostasis, critically shape the neuronal response to hyperexcitation.

Studies on immature neuron cultures and in a clonazepam-induced seizure model in newborn animals have demonstrated that early-life hyperexcitability induces short- and long-term alterations in the expression of glutamate receptor subunits [41,42]. Such alterations can cause an enduring imbalance between excitation and inhibition, thereby establishing a molecular basis for long-term functional impairments.

In contrast to the models described above, the possibility of persistent changes in the glutamatergic system following febrile seizures (FS) remains insufficiently explored. While previous studies have reported changes in a limited number of genes within this system [36,43,44], a comprehensive analysis of the long-term molecular consequences of FS is lacking. This is particularly true for a systematic assessment of the entire spectrum of glutamate receptors and transporters.

Therefore, we aimed to provide a comprehensive characterization of the delayed effects of FS, integrating the analysis of glutamatergic system gene expression with cognitive and emotional outcomes in young adult animals (P45–55). In rodents, this age corresponds to human adolescence [45]—an age when the first clinical manifestations of mental disorders are often observed, occurring, among other things, against the backdrop of puberty [46,47].

To investigate long-term, region-specific changes following FS, we used a rat model of seizures induced at P10 [48]. In young adult animals (P45-55), we assessed: (1) selective neuronal loss in dorsal vs. ventral hippocampal subfields CA1 and CA3; (2) gene expression of glutamatergic targets (NMDA, AMPA, mGluR subunits, EAATs) across limbic and cortical regions; and (3) a spectrum of behavioral outcomes including exploration, anxiety, and memory.

## 2. Materials and Methods

### 2.1. Animals

All experimental procedures utilized male Wistar rats obtained from the animal breeding facility of the Sechenov Institute of Evolutionary Physiology and Biochemistry, Russian Academy of Sciences (St. Petersburg, Russia). The animals were maintained in standard cages under a 12 h light/dark cycle with ad libitum access to food and water. For the study, both control and experimental cohorts were assembled from individuals of different litters to minimize litter-specific effects. All animal handling and experimental protocols were performed in strict adherence to the institutional guidelines of the Sechenov Institute, which are fully aligned with the European Union Directive 2010/63/EU on the protection of animals used for scientific purposes.

**Figure 1:**
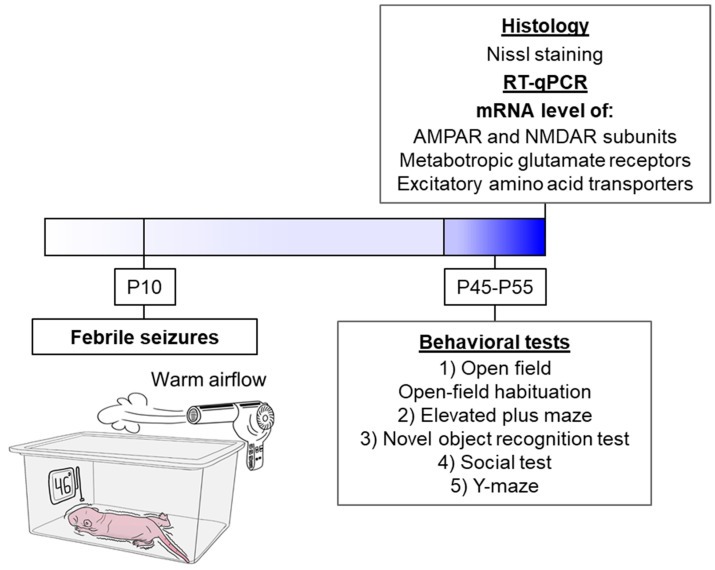
The experimental design. The schematic diagram illustrates the experimental model of FS. The animal is placed in a chamber over which a flow of warm air is created so that a temperature of 46 °C at the bottom of the chamber. Under such conditions, the animal’s body temperature rises, and FS develops (*N* = 17). The littermates utilized as controls were removed from the nest for the same duration but were kept at room temperature (*N* = 23).

The schematic representation delineates the sequence of experimental procedures performed during the study (Figure 1).

Sample size was determined a priori using GraphPad StatMate version 2.00 (GraphPad Software, San Diego, CA, USA) (α = 0.05, power = 0.8) based on effect sizes from prior work. Some values were removed after the experiment if they were identified as outliers. The total number of animals used for the study is shown in Table 1. For the analysis of mRNA expression, eight animals were randomly selected from each group after behavioral testing. Histological analysis was performed on a separate sample of animals.

### 2.2. Febrile Seizure Model

Febrile seizures were provoked in P10 rat pups following a well-characterized hyperthermia paradigm [16]. This developmental stage in rats corresponds to the first year of human life in terms of CNS maturation, and it is at this point that rodents exhibit a seizure threshold and stereotypical seizure progression analogous to human FS [48]. Briefly, pups were individually placed in a glass chamber and subjected to a stream of heated air for 30 min, which maintained an ambient temperature of 46 °C (Figure 1). Core body temperature was monitored rectally at baseline (32–33 °C), at seizure onset (~39 °C), and at two-minute intervals thereafter. Most animals reached a core temperature of 39 °C within 10 min, displaying facial automatisms and unilateral body flexion, followed by myoclonic jerks of the hindlimbs that evolved into generalized clonic convulsions. These behavioral seizures have been electrophysiologically correlated with rhythmic discharges in limbic structures [16]. To prevent hyperthermia exceeding 40 °C, pups were briefly transferred to a cooled surface until their temperature returned to approximately 37 °C, after which they were placed back into the chamber. Only pups exhibiting clonic convulsions for a minimum of 15 min were included in the experimental group. Control littermates were separated from the dam for an equal duration but maintained at room temperature.

### 2.3. Behavioral Testing

A battery of behavioral tests was administered starting at postnatal day 45 (P45). The testing order was identical for all animal cohorts: (1) Open Field with a 3-day habituation protocol, (2) Elevated Plus Maze, (3) Novel Object Recognition, (4) Social Interaction Test, and (5) Y-Maze. Given that these paradigms are considered to induce mild stress, they were performed consecutively, with one test per day.

#### 2.3.1. Open Field Test

Locomotor and anxiety-related behaviors were assessed in a circular open field arena (100 cm diameter, 30 cm high walls) under dim illumination (8 lux). Each rat was introduced to the center of the arena and allowed to explore freely for 3 min while being video-recorded. The resulting videos were analyzed offline with dedicated software (“Tracking” v3.2 and “Field 4” v4.0, Institute of Experimental Medicine, St. Petersburg, Russia). The total distance moved served as an index of general locomotor activity, while the duration of time spent in the central zone (defined as an area with a radius of 50 cm) was used to quantify anxiety-like behavior [49].

#### 2.3.2. Open-Field Habituation

To evaluate habituation to a familiar environment, animals were exposed to the open field arena once daily for three consecutive days at a consistent time. The total distance traveled during each 3 min session was recorded and served as the primary metric for quantifying the reduction in exploratory activity over time.

#### 2.3.3. Elevated Plus Maze

Anxiety-like behavior was further assessed using an elevated plus maze, comprising a central square (10 × 10 cm) from which two open arms and two enclosed arms (each 50 × 10 cm) extended. The apparatus was raised 40 cm above the floor. The closed arms featured 30 cm-high walls with opaque covers, creating a dimly lit environment (1 lux), while the open arms were more brightly illuminated (40 lux). At the start of a trial, a rat was placed in the central area facing an open arm and given 5 min to explore the maze freely. Its behavior was video-tracked, and the time spent in the open versus closed arms was used as the key indicator of anxiety [50].

#### 2.3.4. Novel Object Recognition Test

Exploratory behavior and short-term recognition memory were evaluated using the novel object recognition test, based on established protocols [51,52]. Testing occurred in a Plexiglas chamber (60 × 30 × 40 cm) under video surveillance. Two distinct pairs of toys, selected to preclude inherent preferences and prevent climbing, served as objects. To minimize neophobia and baseline stress, rats were habituated to the empty testing chamber for 24 h prior to the experiment.

The test protocol included two 5 min trials separated by a 1 h inter-trial interval. In Trial 1 (acquisition), two identical objects were positioned in the chamber, and the rat’s exploratory behavior (sniffing, touching, or oral contact) towards them was recorded. The total exploration time in this trial provided a measure of initial exploratory drive.

In Trial 2 (retrieval), one of the familiar objects was replaced with a novel one. The time spent investigating the familiar and the novel object was recorded separately. A discrimination index (DI) was calculated as (T_novel_ − T_familiar_)/(T_novel_ + T_familiar_) to quantify recognition memory, where positive values indicate a preference for novelty.

#### 2.3.5. Social Interaction Test

Social behavior was examined using a resident-intruder paradigm [53]. Resident rats were first habituated to the test cage (60 × 30 × 40 cm) for 24 h to reduce environmental novelty. Subsequently, an unfamiliar, age-matched male Wistar rat (the intruder) was introduced into the home cage for a 5 min session. Social interaction was quantified by measuring the duration of active social behaviors (e.g., sniffing the body or anogenital area of the intruder) displayed by the resident. Aggressive encounters were also timed. Furthermore, the duration of self-grooming by the resident rat was recorded as a potential measure of anxiety in a social context.

#### 2.3.6. Y-Shaped Maze Spontaneous Alternation Test

Spatial working memory was evaluated in a Y-maze consisting of three identical arms (50 × 10 cm each) with 30 cm-high opaque walls. Each rat was placed in the central area and allowed to freely explore the maze for 8 min. An arm entry was recorded when all four paws crossed the threshold. Spontaneous alternation behavior, defined as sequential entry into three different arms (e.g., 1-2-3, 2-3-1), was considered a correct choice. Working memory performance was quantified using the alternation coefficient (CA), calculated as follows: CA = N_right_/(N_total_ − 2), where N_right_ represents the number of correct entries into a new arm, and Ntotal is the total number of arm inputs, a measure of total locomotor activity.

### 2.4. mRNA Expression Analysis

Brain isolation for subsequent RT-qPCR was performed on day 55 of rat life. Based on the rat brain atlas [54], the following brain regions were dissected using an OTF5000 cryostat microtome (Bright Instrument, Luton, UK): the dorsal and ventral hippocampus, medial prefrontal cortex, and temporal cortex. Total RNA was extracted from these regions using ExtractRNA reagent (Evrogen, Moscow, Russia) according to the manufacturer’s protocol and stored in 75% ethanol at −20 °C.

RNA samples were treated with RQ1 DNase (Promega, Madison, WI, USA) to eliminate genomic DNA contamination. RNA concentration and purity were verified spectrophotometrically. First-strand cDNA was synthesized from 1 μg of total RNA using a mix of oligo-dT and random 9-mer primers (DNA-Synthesis, Moscow, Russia) with M-MLV reverse transcriptase (Evrogen).

Quantitative PCR was performed in 6 μL reactions containing diluted cDNA, TaqM polymerase (Alkor Bio, St. Petersburg, Russia), 3.5 mM MgCl_2_, and gene-specific primers/probes (Table 2; DNA-Synthesis). Amplification was performed using a CFX384 Real-Time System (Bio-Rad, Hercules, CA, USA) with four technical replicates per sample. The multiplexes used for housekeeping genes have been described previously [55]. The following multiplexes for the genes of interest were used in this study: *Grin1 + Grin2a*, *Grin2b + Gria1 + Gria2*, *Grm1 + Grm3 + Grm5*, *Grm2 + Grm7 + Grm8*, *Slc1a1 + Slc1a3*, except for *Grm4* and *Slc1a2*, which were run in singleplex.

The relative expression of the genes was evaluated using the 2^−ΔΔ^Ct method [56]. The most stably expressed genes for each structure were selected using the RefFinder online tool (https://www.ciidirsinaloa.com.mx/RefFinder-master/ (accessed on 16 June 2025)). Normalization was performed using the following genes: *Rpl13a*, *Pgk1*, *Ywhaz* for dorsal hippocampus; *Ppia*, *Gapdh* for ventral hippocampus; *Actb*, *Gapdh* for temporal cortex; *Pgk1*, *Ywhaz* for medial prefrontal cortex. For the selection of reference genes, we relied on our previous findings from the same model [57]. The detailed results and stability rankings from the RefFinder analysis are provided in Table A1, Table A2, Table A3 and Table A4.

**Table 2:** Primer and probe sequences for RT-qPCR.

Gene Symbol RefSeq Accession Number	Forward Primer, Reverse Primer, and Probe Sequences (5′→3′)	Final Concentrations, nM	References
*Actb* NM_031144	TGTCACCAACTGGGACGATA GGGGTGTTGAAGGTCTCAAA FAM-CGTGTGGCCCCTGAGGAGCAC-BHQ1	200 200	[58] (primers) [55] (probe)
*Gapdh* NM_017008	TGCACCACCAACTGCTTAG GGATGCAGGGATGATGTTC R6G-ATCACGCCACAGCTTTCCAGAGGG-BHQ2	200 100	[59]
*B2m* NM_012512	TGCCATTCAGAAAACTCCCC GAGGAAGTTGGGCTTCCCATT ROX-ATTCAAGTGTACTCTCGCCATCCACCG-BHQ1	200 100	[60]
*Rpl13a* NM_173340	GGATCCCTCCACCCTATGACA CTGGTACTTCCACCCGACCTC FAM-CTGCCCTCAAGGTTGTGCGGCT-BHQ1	200 100	[61] (primers) [55] (probe)
*Sdha* NM_130428	AGACGTTTGACAGGGGAATG TCATCAATCCGCACCTTGTA R6G-ACCTGGTGGAGACGCTGGAGCT-BHQ2	200 100	[62] (primers) [55] (probe)
*Ppia* NM_017101	AGGATTCATGTGCCAGGGTG CTCAGTCTTGGCAGTGCAGA ROX-CACGCCATAATGGCACTGGTGGCA-BHQ1	200 100	[63]
*Hprt1* NM_012583	TCCTCAGACCGCTTTTCCCGC TCATCATCACTAATCACGACGCTGG FAM-CCGACCGGTTCTGTCATGTCGACCCT-BHQ1	200 100	[64] (primers) [55] (probe)
*Pgk1* NM_053291	ATGCAAAGACTGGCCAAGCTAC AGCCACAGCCTCAGCATATTTC R6G-TGCTGGCTGGATGGGCTTGGA-BHQ2	200 100	[65] (primers) [55] (probe)
*Ywhaz* NM_013011	GATGAAGCCATTGCTGAACTTG GTCTCCTTGGGTATCCGATGTC ROX-TGAAGAGTCGTACAAAGACAGCACGC-BHQ1	200 100	[65] (primers) [55] (probe)
*Grm1* NM_001114330.1	GGGAATGCCAAGAAGAGGCAG CTGCAGTGTGGGGGTTTTCAA FAM-GCCCAGCAGCCAGTGTCCGTCGGC-BHQ1	400 200	[66]
*Grm2* NM_001105711.1	TCCAGTGATTATCGGGTGCAG AACTTGGGTGCAAAGAGGCA FAM-TGCGTGTCCGTCAGCCTCAGTGGCT-BHQ1	200 100	[66]
*Grm3* NM_001105712.1	CAGGAGTTGACGGTGCGAG GCCTGTCCTTCAGATAAGGGAG ROX-TCGGTGACGGGCTCTTTCAGCCCAA-BHQ2	200 200	[66]
*Grm4* NM_022666.1	GGCAGTGCGAGCAGCTAAGG CCGGTCACTCCTACCAACCG FAM-CTCCCTGAGCTCCCCCGGAGCAGC-BHQ1	200 150	[66]
*Grm5* NM_017012.1	ATGCATGTAGGAGACGGCAA TTTCCGTTGGAGCTTAGGGTTT HEX-CGTCCGCTGCCAGCAGATCCAGCA-BHQ2	400 200	[67] (primers) [66] (probe)
*Grm7* NM_031040.1	CCAGACAACAAACACAACCAACC GCGTTCCCTTCTGTGTCTTCTTC HEX-TGCAGTGGGGCAAAGGAGTCCGAG-BHQ2	200 100	[68] (primers) [66] (probe)
*Grm8* NM_022202.1	TCATCGGGCACTGGACAAAT CACGGTTTTCTTCCTCTCCCC ROX-TGTCTGCAGCCTGCCGTGCAAGCCC-BHQ2	300 100	[66]
*Grin1* NM_017010	GTTCTTCCGCTCAGGCTTTG AGGGAAACGTTCTGCTTCCA FAM-CGGCATGCGCAAGGACAGCC-BHQ1	200 100	[69]
*Grin2a* NM_012573	GCTACACACCCTGCACCAATT CACCTGGTAACCTTCCTCAGTGA FAM-TGGTCAATGTGACTTGGGATGGCAA-BHQ1	200 100	[70]
*Grin2b* NM_012574	CCCAACATGCTCTCTCCCTTAA CAGCTAGTCGGCTCTCTTGGTT FAM-GACGCCAAACCTCTAGGCGGACAG-BHQ1	200 100	[70]
*Gria1* NM_031608	TCAGAACGCCTCAACGCC TGTAGTGGTACCCGATGCCA ROX-TCCTGGGCCAGATCGTGAAGCTAGAAAA-BHQ1	200 100	[71]
*Gria2* NM_017261	CAGTGCATTTCGGGTAGGGA TGCGAAACTGTTGGCTACCT FAM-TCGGAGTTCAGACTGACACCCCA-BHQ1	200 100	[71]
*Slc1a1* NM_013032.3	CCTGCATCCCTCATCCCAC CTCCTACCACGATGCCCAGTA HEX-CCGCCGCGCTCCCCGATTCC-BHQ2	200 100	[72]
*Slc1a2* NM_001035233.1	CCAGTGCTGGAACTTTGCCT TAAAGGGCTGTACCATCCAT FAM-AGCGTGTGACCAGATTCGTCCTCCCA-BHQ1	200 150	[73] (primers) [74] (probe)
*Slc1a3* NM_019225.2	GCGCTGTCATTGTGGGTACA CAGAAGCTCCCCAGGAAAGG Cy5-CCTTGGATTTGCCCTCCGACCGT-BHQ3	200 100	[75]

### 2.5. Histology

#### 2.5.1. Preparation of Brain Tissue for Histological Examination

For histological examination, P55 rats were deeply anesthetized and transcardially perfused with ice-cold PBS followed by 4% paraformaldehyde. Brains were post-fixed in 4% PFA for 48 h at 4 °C, cryoprotected in 30% sucrose, and rapidly frozen in chilled isopentane. Serial 20-μm coronal sections encompassing the dorsal and ventral hippocampus were cut on a cryostat. To control for potential lateralization, one hemisphere was randomly chosen per animal. Four sections from each hippocampal region, spaced 100 μm apart, were mounted on adhesive slides and stored at −20 °C until staining.

#### 2.5.2. Staining of Sections Using the Nissl Method

**Figure 2:**
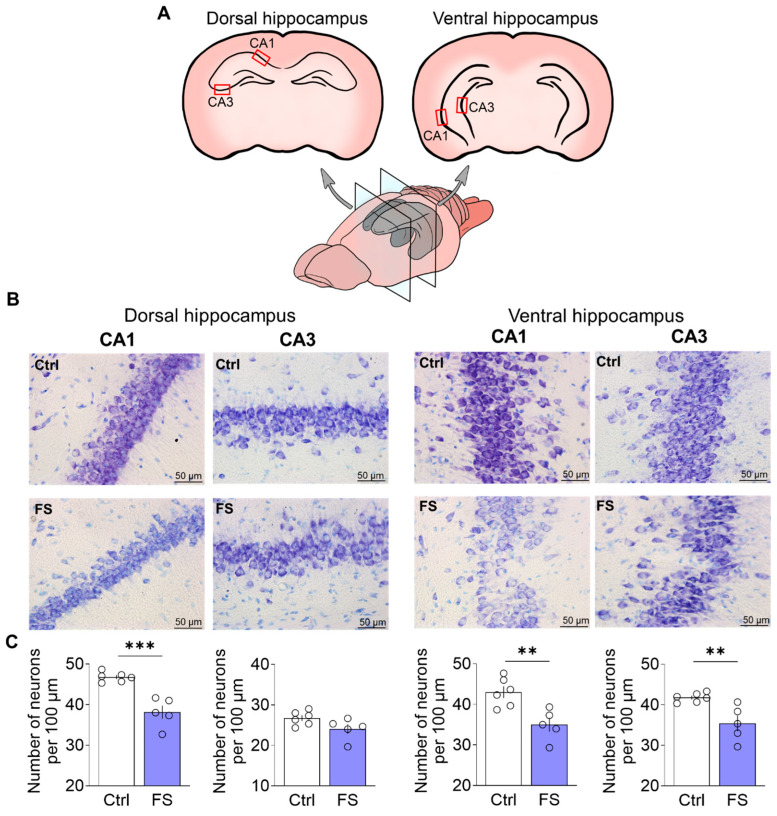
(**A**) Schematic representation of dorsal and ventral hippocampal sections. The regions of the brain in which neurons were quantified are indicated. (**B**) CA1 and CA3 areas of the dorsal and ventral hippocampus of animals in the control (*N* = 6) and experimental groups (*N* = 5). (**C**) Diagrams illustrating the number of neurons per 100 µm of cell layer length in the CA1 and CA3 areas of the dorsal hippocampus and in the CA1 and CA3 areas of the ventral hippocampus. The dots represent the individual values for each rat. Student’s test: **—*p* < 0.01, ***—*p* < 0.001.

Nissl staining was performed as previously described in detail [76]. Images using a ×40 lens were obtained using a Leica AF7000 light microscope (Leica Microsystems, Wetlar, Germany). Quantitative analysis was performed visually in 100 µm of cell layer length in the CA1 and CA3 regions of the dorsal and ventral hippocampus in photographs using ImageJ 1.53k (U.S. National Institutes of Health, Bethesda, MD, USA). Cell counting was conducted by an experimenter blinded to the experimental groups. Sampling areas were consistently identified using standardized anatomical landmarks as illustrated in Figure 2A.

### 2.6. Statistical Analysis

Statistical analysis was conducted using the GraphPad Prism 8 software (GraphPad Software, San Diego, CA, USA). Potential outliers were identified with Dixon’s *Q*-test, and data distribution was assessed for normality using the Kolmogorov–Smirnov test. Based on distribution characteristics, we applied either parametric tests (Student’s *t*-test, mixed ANOVA with Sidak’s post hoc comparison) or non-parametric alternatives (Mann–Whitney *U* test). Statistical significance was defined as *p* < 0.05.

For qPCR data, results are displayed as box plots showing individual data points, quartiles, and median values. Multiple comparison correction was not applied because each gene was analyzed as an independent hypothesis. Behavioral data are expressed as means ± SEM for normally distributed parameters or medians with interquartile ranges for non-normally distributed measures.

Effect sizes were calculated as Cohen’s *d* for *t*-tests and *η^2^* for ANOVA, with conventional thresholds for interpretation: 0.2 (small), 0.5 (medium), 0.8 (large), 1.5 (very large), and 2.5 (huge); for η^2^: 0.01 (small), 0.06 (medium), and 0.14 (large).

## 3. Results

### 3.1. Febrile Seizures Provoke Neuronal Loss in the Rat Hippocampus

To determine whether FS led to neuronal loss, we quantified neurons in the hippocampus of adult rats (P55). Given the functional differentiation along the dorsoventral axis [17], we separately analyzed neuronal counts in the CA1 and CA3 subfields of both the dorsal and ventral hippocampus. For this purpose, we used Nissl staining followed by quantitative analysis (Figure 2).

In the dorsal hippocampus, a significant difference in the number of neurons between control animals and animals after FS was observed in the CA1 region (CA1: control: 46.8 ± 0.5 neurons per 100 μm, 95% CI [45.5, 48.0], *N* = 6 rats; FS: 38.1 ± 1.6, 95% CI [33.7, 42.6], *N* = 5, *t* = 5.6, *p* < 0.001, *d* = 3.39). However, there were no differences between the groups in the CA3 region (CA3: control: 26.7 ± 0.7, 95% CI [24.9, 28.5], FS: 24.1 ± 1.2, 95% CI [20.8, 27.4], *t* = 2.01, *p* = 0.08).

In contrast to the dorsal hippocampus, FS induced significant neuronal loss in both the CA1 and CA3 regions of the ventral hippocampus (CA1: control: 43.0 ± 1.4, 95% CI [39.3, 46.7], FS: 35.0 ± 1.7, 95% CI [30.3, 39.7], *t* = 3.64, *p* = 0.005, *d* = 2.20; CA3: control: 41.8 ± 0.5, 95% CI [40.6, 43.0], FS: 35.4 ± 1.9, 95% CI [30.0, 40.8], *t* = 3.5, *p* = 0.007, *d* = 2.12).

### 3.2. Changes in Glutamate Receptors and Excitatory Amino Acid Transporters Gene Expression in Rat Brain After Febrile Seizures

We examined gene expression of NMDA and AMPA receptor subunits, mGluRs, as well as EAATs in the dorsal and ventral hippocampus, temporal and medial prefrontal cortex of rats that had undergone FS. The analysis was performed at P55.

**Figure 3:**
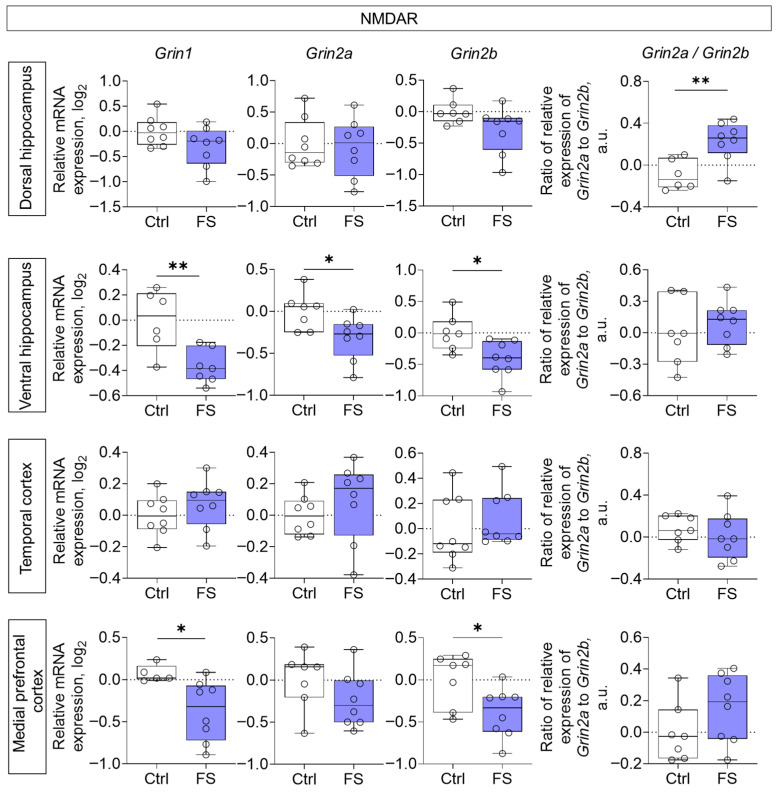
Relative gene expression of NMDA receptor subunits (*Grin1*, *Grin2a*, *Grin2b*) and ratio of Grin2a/Grin2b mRNA production in the dorsal and ventral hippocampus, temporal and medial prefrontal cortex of rats in a model of FS at P55. Ctrl–animals weaned for the time of modeling seizures from the nest, but kept in normothermic conditions (N = 6–8), FS–animals after modeling FS at P10 (N = 7–8). Data are presented as individual values with minimum and maximum values (error whiskers), sample median (horizontal line), and first and third quartiles. Student’s test: *—*p* < 0.05, **—*p* < 0.01.

Decreased expression of all NMDA receptor subunits studied (*Grin1*, *Grin2a*, *Grin2b*) was noted in the ventral region of the hippocampus (*Grin1*: *t* = 3.4, *p* = 0.006, *d* = 1.91; *Grin2a*: *t* = 2.5, *p* = 0.026, *d* = 1.31; *Grin2b*: *t* = 2.8, *p* = 0.015, *d* = 1.46), while *Grin1* and *Grin2b* gene expression was reduced in the medial prefrontal cortex (*Grin1*: *t* = 2.6, *p* = 0.023, *d* = 1.50; *Grin2b*: *t* = 2.5, *p* = 0.027, *d* = 1.23) of rats after FS (Figure 3). Additionally, we calculated the ratio of *Grin2a*/*Grin2b* mRNA production. This ratio reflects the balance between *Grin2a* and *Grin2b* mRNA, which affects the formation of receptor composition, and thus, the functional activity of the glutamatergic synapse and synaptic plasticity processes [77,78]. We found that it was increased in the dorsal hippocampus (*t* = 3.5, *p* = 0.005, *d*= 1.86).

**Figure 4:**
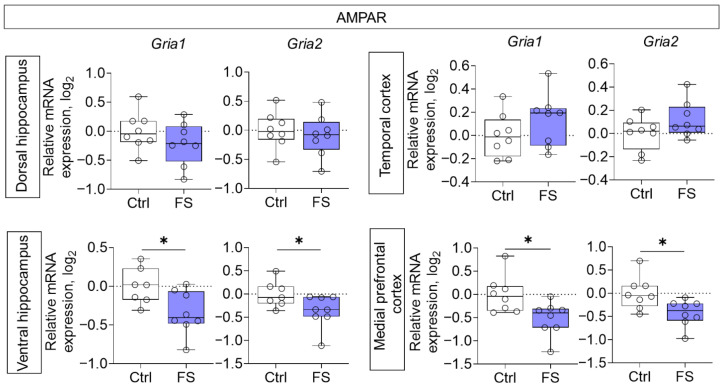
Relative gene expression of AMPA receptor subunits (*Gria1*, *Gria2*) in the dorsal and ventral hippocampus, temporal and medial prefrontal cortex of rats in a model of FS at P55. Ctrl–animals weaned for the time of modeling seizures from the nest, but kept in normothermic conditions (N = 7–8), FS–animals after modeling FS at P10 (N = 7–8). Data are presented as individual values with minimum and maximum values (error whiskers), sample median (horizontal line), and first and third quartiles. Student’s test: *—*p* < 0.05.

Decreased mRNA production of genes of both investigated AMPA receptor subunits (*Gria1*, *Gria2*) was detected in the ventral region of the hippocampus (*Gria1*: *t* = 2.5, *p* = 0.025, *d* = 1.31; *Gria2*: *t* = 2.2, *p* = 0.044, *d* = 1.15) and in the medial prefrontal cortex (*Gria1*: *t* = 2.7, *p* = 0.017, *d* = 1.35; *Gria2*: *t* = 2.7, *p* = 0.019, *d* = 1.33) (Figure 4).

**Figure 5:**
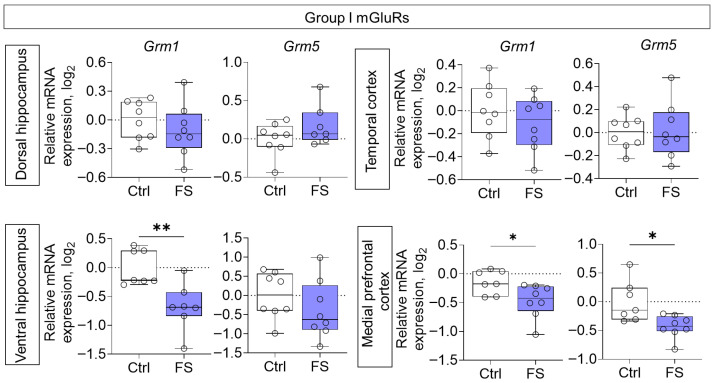
Relative gene expression of the group I mGluRs (*Grm1*, *Grm5*) in the dorsal and ventral hippocampus, temporal and medial prefrontal cortex of rats in a model of FS at P55. Ctrl–animals weaned for the time of modeling seizures from the nest, but kept in normothermic conditions (N = 7–8), FS–animals after modeling FS at P10 (N = 7–8). Data are presented as individual values with minimum and maximum values (error whiskers), sample median (horizontal line), and first and third quartiles. Student’s test: *—*p* < 0.05; Mann–Whitney test: **—*p* < 0.01.

Reduced gene expression of members of group I mGluRs was also observed in the ventral hippocampal region (*Grm1*: Mann–Whitney test U_7,7_ = 4, *p* = 0.007) and medial prefrontal cortex (*Grm1*: *t* = 2.5, *p* = 0.029, *d* = 1.27; *Grm5*: *t* = 2.9, *p* = 0.012, *d* = 1.52) of rats after FS (Figure 5).

**Figure 6:**
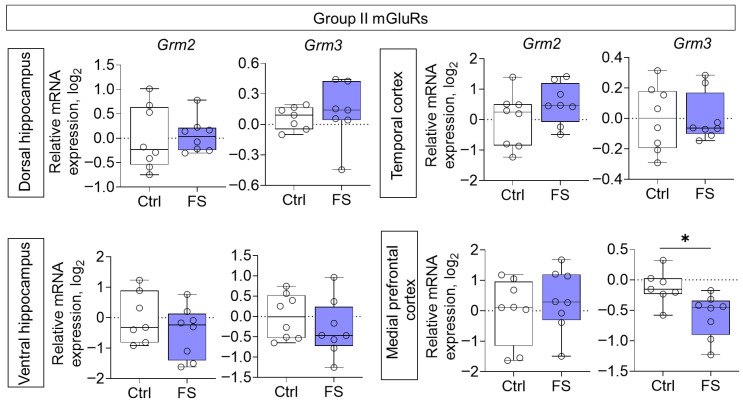
Relative gene expression of the group II mGluRs (*Grm2*, *Grm3*) in the dorsal and ventral hippocampus, temporal and medial prefrontal cortex of rats in a model of FS at P55. Ctrl–animals weaned for the time of modeling seizures from the nest, but kept in normothermic conditions (N = 6–8), FS–animals after modeling FS at P10 (N = 7–8). Data are presented as individual values with minimum and maximum values (error whiskers), sample median (horizontal line), and first and third quartiles. Student’s test: *—*p* < 0.05.

**Figure 7:**
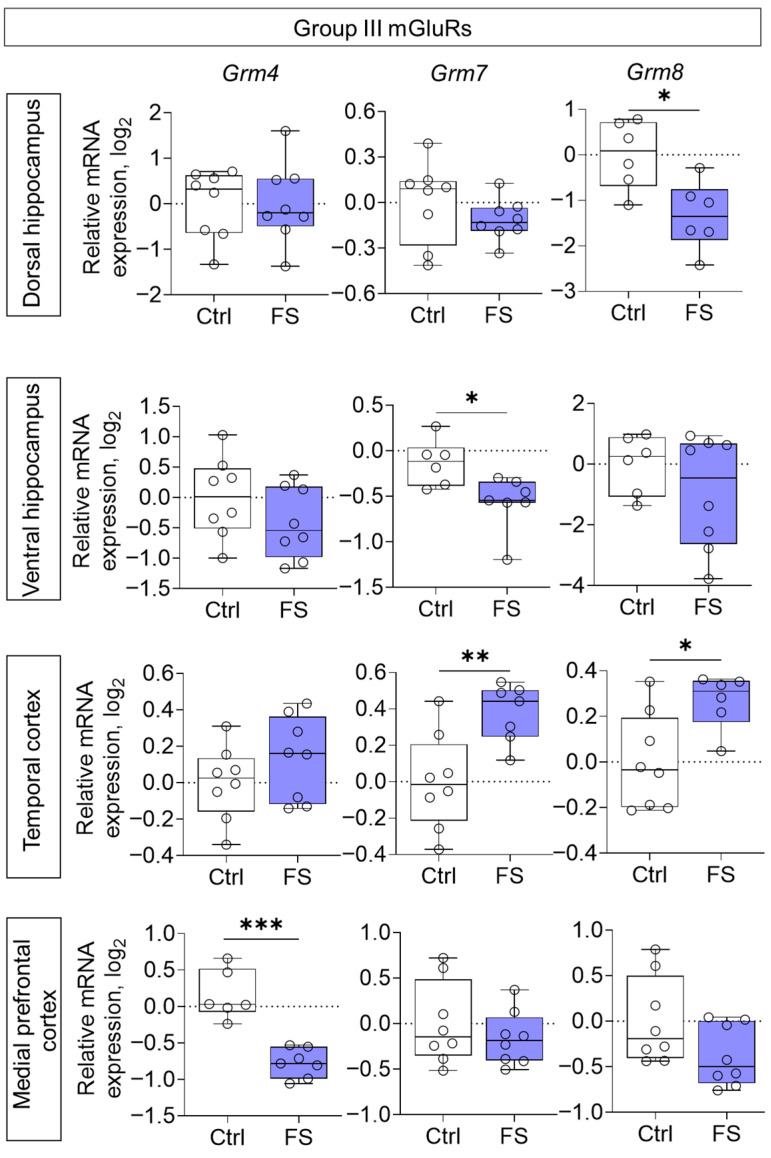
Relative gene expression of the group III mGluRs (*Grm4*, *Grm7*, *Grm8*) in the dorsal and ventral hippocampus, temporal and medial prefrontal cortex of rats in a model of FS at P55. Ctrl–animals weaned for the time of modeling seizures from the nest, but kept in normothermic conditions (N = 6–8), FS–animals after modeling FS at P10 (N = 6–8). Data are presented as individual values with minimum and maximum values (error whiskers), sample median (horizontal line), and first and third quartiles. Mann–Whitney test: *—*p* < 0.05 (*Grm7* gene expression in the ventral hippocampus); the Student’s test: *—*p* < 0.05, **—*p* < 0.01, ***—*p* < 0.001.

The changes in mRNA production of group II mGluRs were less pronounced: we found a decrease in *Grm3* gene expression only in the medial prefrontal cortex (*t* = 2.8, *p* = 0.015, *d* = 1.45) (Figure 6). By contrast, the expression of group III metabotropic glutamate receptor genes was altered in all of the rat brains examined after they had suffered FS at an early age (Figure 7). In particular, the production of *Grm4* mRNA was reduced in the medial prefrontal cortex (*t* = 6.2, *p* < 0.001, *d* = 3.44). Meanwhile, *Grm7* gene expression changed in different directions: it decreased in the ventral hippocampus (Mann–Whitney test U_6,7_ = 4, *p* = 0.014) and increased in the temporal cortex (*t* = 3.3, *p* = 0.006, *d* = 1.71). Similar changes in the temporal cortex were also seen with *Grm8* gene expression (*t* = 2.8, *p* = 0.017, *d* = 1.50). However, *Grm8* mRNA production decreased in the dorsal region of the hippocampus (*t* = 3.1, *p* = 0.011, *d* = 1.80).

**Figure 8:**
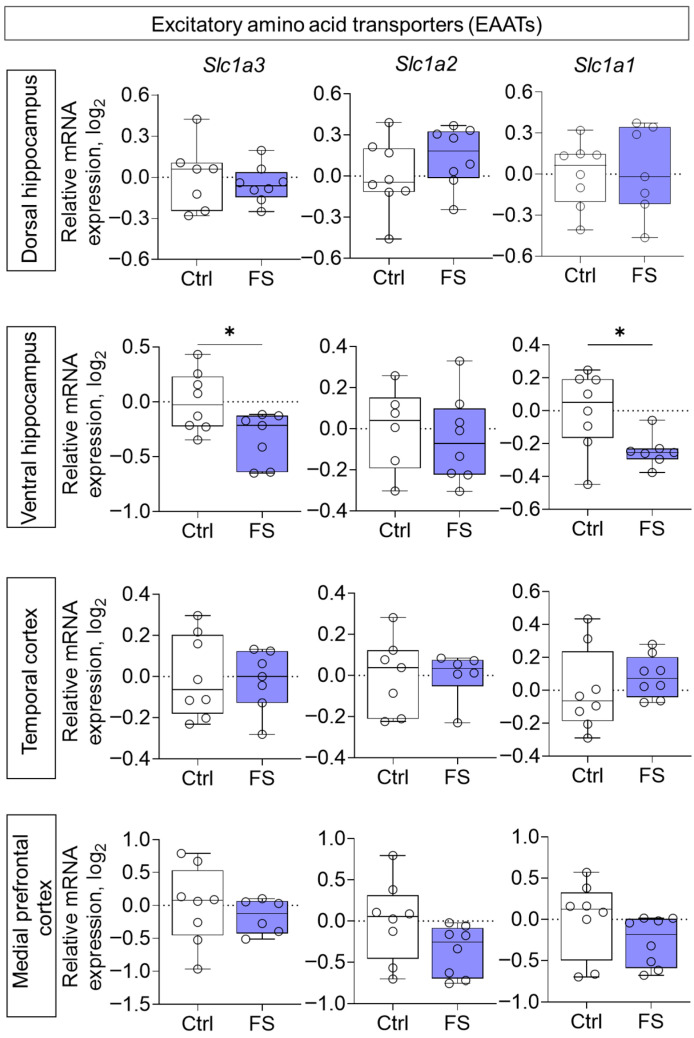
Relative gene expression of the excitatory amino acid transporters (*Slc1a3*, *Slc1a2*, *Slc1a1*) in the dorsal and ventral hippocampus, temporal and medial prefrontal cortex of rats in a model of FS at P55. Ctrl–animals weaned for the time of modeling seizures from the nest, but kept in normothermic conditions (N = 7–8), FS–animals after modeling FS at P10 (N = 6–8). Data are presented as individual values with minimum and maximum values (error whiskers), sample median (horizontal line), and first and third quartiles. Student’s test: *—*p* < 0.05.

Furthermore, the expression of genes encoding EAATs was studied. A decline in expression was observed following FS, exclusively in the ventral hippocampus (Figure 8, *Slc1a3* (encoding EAAT1): *t* = 2.5, *p* = 0.026, *d* = 1.31; *Slc1a1* (encoding EAAT3): *t* = 2.6, *p* = 0.024, *d* = 1.33).

Thus, long-term changes in the expression iGluRs, mGluRs and EAATs are observed in the rat brain following neonatal FS. The most pronounced alterations were observed in the medial prefrontal cortex and ventral hippocampus. Such region-specific changes in the expression of genes encoding components of the glutamatergic system may underlie a variety of behavioral disorders. So, in this study, we evaluated the behavior of adult rats that had undergone FS.

### 3.3. Behavioral Alterations

#### 3.3.1. Decline in Locomotor Activity and Exploratory Behavior

**Figure 9:**
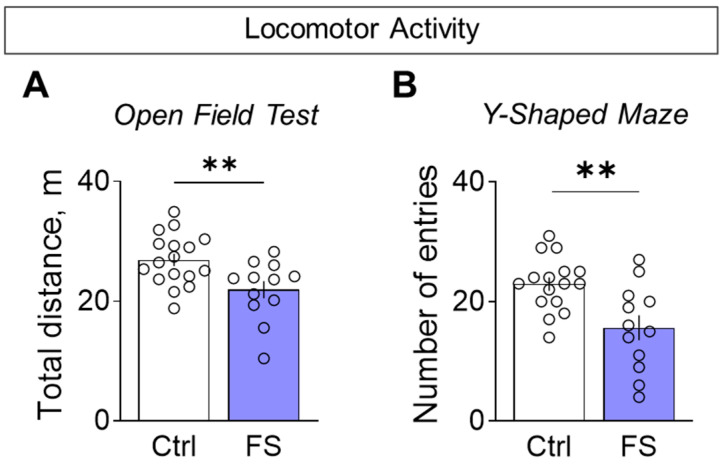
Locomotor activity assessment. (**A**) Total distance traveled in the open field test. (**B**) The number of visited arms in the Y-shaped maze. Ctrl–animals weaned for the time of modeling seizures from the nest, but kept in normothermic conditions (*N* = 16–17), FS–animals after modeling FS at P10 (*N* = 12). Data are presented as means and standard errors of the mean. The circles show the individual values for each rat. Student’s test: **—*p* < 0.01.

Rats with a history of FS were less active when exploring novel environments. This hypolocomotion was consistent across tests: they traveled a shorter distance in the open field (Figure 9A, control: 26.9 ± 1.1 m, 95% CI [24.7, 29.1], *N* = 17; FS: 21.9 ± 1.5 m, 95% CI [18.7, 25.1], *N* = 12; *t* = 2.9, *p* = 0.008, *d* = 1.01) and entered fewer arms in the Y-maze (Figure 9B, control: 22.9 ± 1.1 entries, 95% CI [20.5, 25.4], *N* = 16; FS: 15.6 ± 2.1 entries, 95% CI [11.0, 20.2], *N* = 12; *t* = 3.3, *p* = 0.003, *d* = 1.26) compared to control.

**Figure 10:**
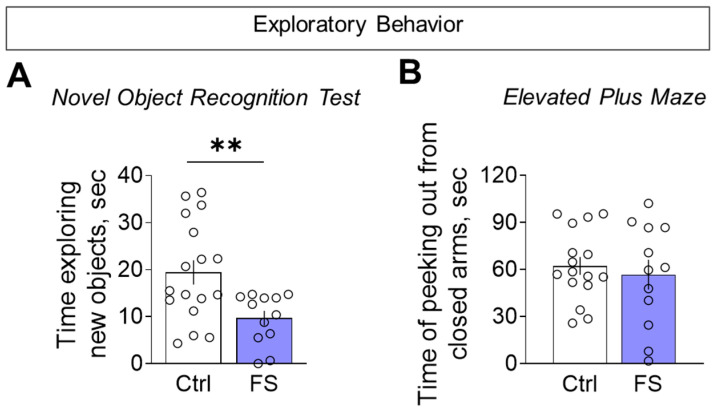
Exploratory behavior assessment. (**A**) Time exploring unfamiliar identical objects when they were first presented in the novel object recognition test. (**B**) Time of peeking out from closed arms in the elevated plus maze. Ctrl–animals weaned for the time of modeling seizures from the nest, but kept in normothermic conditions (*N* = 16–17), FS–animals after modeling FS at P10 (*N* = 12). Data are presented as means and standard errors of the mean. The circles show the individual values for each rat. Student’s test: **—*p* < 0.01.

To determine whether reduced motor activity reflected a broader decline in exploration, we directly assessed exploratory behavior. In the novel object recognition test, FS rats investigated the unfamiliar objects for less time than controls during the initial presentation (Figure 10A, control: 19.4 ± 2.6 s, 95% CI [14.0, 24.9], *N* = 17; FS: 9.7 ± 1.6 s, 95% CI [6.2, 13.1], *N* = 12; *t* = 2.9, *p* = 0.007, *d*= 1.10). We found no difference between the groups in the elevated plus maze for the time spent head-dipping from the closed arms, a behavior used to assess exploratory activity (Figure 10B, control: 62.3 ± 5.7 s, 95% CI [50.2, 74.3], *N* = 16; FS: 56.7 ± 9.5 s, 95% CI [35.7, 77.7], *N* = 12; *t* = 0.5, *p* = 0.6). This confirmed a specific deficit in exploratory drive, which was not solely attributable to general hypolocomotion.

#### 3.3.2. Cognitive Functions

**Figure 11:**
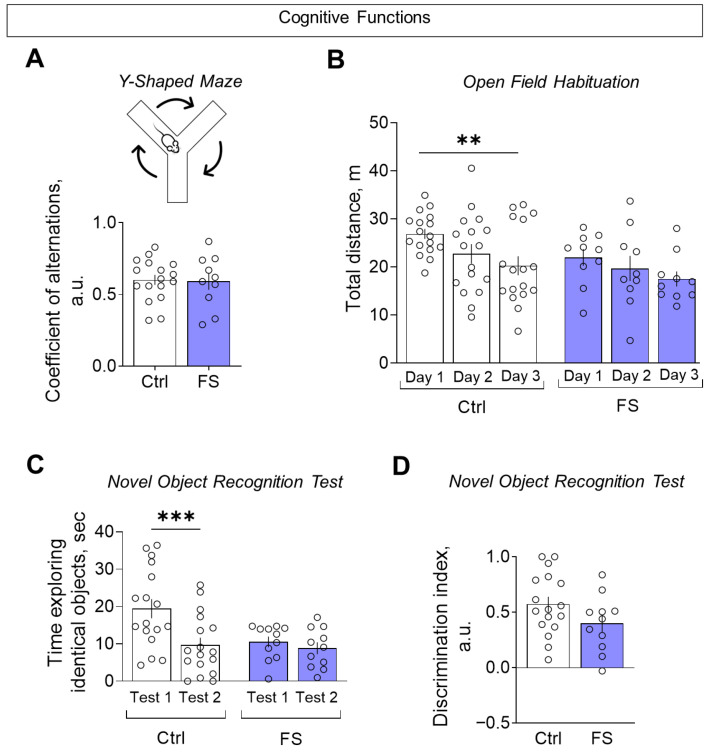
Assessment of cognitive functions. (**A**) Coefficient of alternations in the Y-shaped maze. (**B**) Distance traveled in the open field test over 3 consecutive days. (**C**) The time taken to examine the same object when first presented (test 1) and one hour later (test 2) in the novel object recognition test. (**D**) Discrimination index for assessing preference for a new object compared to a familiar object in the novel object recognition test. Ctrl–animals weaned for the time of modeling seizures from the nest, but kept in normothermic conditions (*N* = 17), FS–animals after modeling FS at P10 (*N* = 10–12). Data are presented as means and standard errors of the mean. The circles show the individual values for each rat. Sidak’s post hoc test: **—*p* < 0.01, ***—*p* < 0.001.

The assessment of cognitive functions was conducted using three distinct tests: the Y-maze, the open field habituation and the novel object recognition test. In the Y-shaped maze, we did not observe any changes in the coefficient of alternation (Figure 11A, control: 0.60 ± 0.04, 95% CI [0.53, 0.68], *N* = 17; FS: 0.59 ± 0.06, 95% CI [0.46, 0.72], *N* = 10; *t* = 0.16, *p* = 0.87), suggesting no impairment of spatial working memory.

In the open field habituation test, FS rats failed to reduce their exploratory activity over three consecutive days, unlike controls, indicating impaired habituation (Figure 11B, mixed ANOVA F(FS)_2,50_ = 6.55, *p* = 0.003, η^2^ = 0.21). A similar deficit in habituation was seen in the novel object recognition test: FS rats spent similar time exploring a familiar object upon re-presentation, while controls showed a significant decrease (Figure 11C, mixed ANOVA F(FS × time)_1,26_ = 5.28, *p* = 0.03, η^2^ = 0.29). Finally, when assessed by the discrimination index (where +1 indicates a total preference for the novel object), there was no significant difference between the groups (Figure 11D, control: 0.57 ± 0.07, 95% CI [0.30, 0.72], *N* = 17; FS: 0.40 ± 0.08, 95% CI [0.23, 0.57], *N* = 11; *t* = 1.67, *p* = 0.11), indicating that the ability to discriminate novelty per se was intact.

#### 3.3.3. Social Interaction

**Figure 12:**
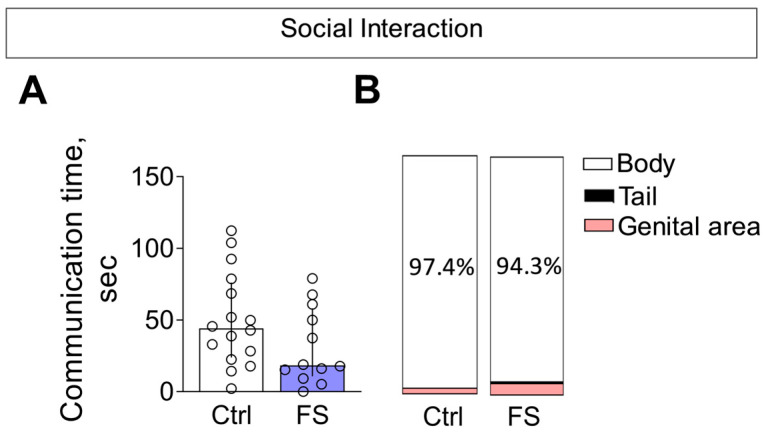
Social interaction. (**A**) Time of communication. Ctrl–animals weaned for the time of modeling seizures from the nest, but kept in normothermic conditions (*N* = 16), FS–animals after modeling FS at P10 (*N* = 12). Data are presented as median and interquartile range. The circles show the individual values for each rat. (**B**) Structure of communicative behavior.

In the social interaction test, we observed no alterations in the total time spent in social interaction between rats with a history of FS and control animals (Figure 12A, Mann–Whitney test U_16,12_ = 63, *p* = 0.13).

The composition of communicative behaviors was also similar between groups (Figure 12B). In both control and FS rats, the majority of social investigation was directed toward the body of the unfamiliar conspecific. Notably, the time spent sniffing the genital area—a behavior associated with higher confidence—was low (<5%) and did not differ between groups. Furthermore, we detected no differences in the occurrence of aggressive behaviors (Mann–Whitney test U_16,12_ = 79, *p* = 0.4).

#### 3.3.4. Anxiety Levels

**Figure 13:**
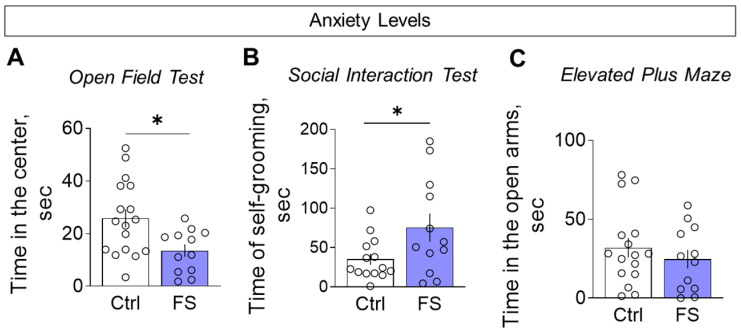
Assessment of anxiety levels. (**A**) Time in the central zone in the open field test. (**B**) Time spent on grooming in the social interaction test. (**C**) Time in open arms in the elevated plus maze. Ctrl–animals weaned for the time of modeling seizures from the nest, but kept in normothermic conditions (*N* = 14–17), FS–animals after modeling FS at P10 (*N* = 12). Data are presented as M ± SEM. The circles show the individual values for each rat. Student’s test: *—*p* < 0.05.

We assessed anxiety-like behavior using multiple parameters. Compared to controls, FS rats spent less time in the center of the open field (Figure 13A, control: 25.8 ± 3.4 s, 95% CI [18.5, 33.1], *N* = 17; FS: 13.5 ± 2.4 s, 95% CI [8.4, 18.7], *N* = 12; *t* = 2.6, *p* = 0.012, *d* = 1.02) and more time engaged in self-grooming during the social interaction test (Figure 13B, control: 35.0 ± 7.0 s, 95% CI [19.8, 50.2], *N* = 14; FS: 75.3 ± 17.8 s, 95% CI [36.0, 114.5], *N* = 12; *t* = 2.2, *p* = 0.036, *d* = 0.87), collectively indicating a heightened anxiety-like state.

However, no significant differences were observed between the groups in time spent in the open arms (Figure 13C, control: 32.0 ± 6.1 s, 95% CI [18.9, 45.0], *N* = 16; FS: 24.7 ± 5.9 s, 95% CI [11.7, 37.7], *N* = 12; *t* = 0.8, *p* = 0.41).

Consequently, mature animals that have experienced prolonged FS at an early age exhibit a specific behavioral phenotype characterized by increased anxiety, decreased motor activity and exploratory behavior, and impaired habituation to familiar environments, without impairing working memory, recognition of new objects and social behavior.

## 4. Discussion

Febrile seizures (FS) are a common neurological disorder in early childhood, yet considerable debate persists regarding the long-term histopathological, neurochemical, and behavioral consequences of complex FS forms [79,80].

Both clinical and experimental evidence indicates that the profound brain hyperactivation during severe neonatal FS can result in cognitive deficits [34]. A leading hypothesis posits that this hyperactivation triggers excitotoxic and neuroinflammatory cascades, which disrupt normal developmental trajectories and produce long-term alterations, frequently linked to hippocampal dysfunction [76,81,82,83]. However, these studies did not account for the structural-functional heterogeneity of the hippocampus—particularly the distinct roles of its dorsal and ventral segments in spatial memory and emotional regulation, respectively [84]. Furthermore, the neurochemical mechanisms underlying FS-associated neuropsychiatric disorders remain poorly understood.

This study provides a comprehensive analysis of the long-term consequences in young adult rats (P45-55) following experimental FS induced at a developmentally critical period (P10). We report an uneven pattern of neuronal loss, alongside significant and regionally distinct alterations in the expression of genes encoding ionotropic (NMDA, AMPA) and mGluRs, as well as EAATs in the dorsal and ventral hippocampus, temporal cortex, and medial prefrontal cortex. These molecular changes were accompanied by a spectrum of behavioral abnormalities, including impaired cognitive function (exploratory behavior and memory), diminished motor activity in a novel environment, and heightened anxiety.

### 4.1. The Histopathological Changes

Our data confirm earlier reports of hippocampal neuronal loss following prolonged FS [85] and extend these findings by revealing a specific topographical pattern of vulnerability. Excitotoxicity and neuroinflammation represent two principal mechanisms likely mediating seizure-induced neuronal death. Fever directly enhances neuronal excitability [27] and hyperthermia-induced seizures in immature rats involve amplified glutamate-triggered intracellular Ca^2+^ release—a process whose pharmacological inhibition attenuates FS severity [86]. Inflammatory processes also play a critical role in modulating glutamate dynamics during FS. Fever-associated elevations in pro-inflammatory cytokines, particularly IL-1β, have been shown to promote glutamate release from both neurons and glial cells. These effects lead to accumulation of extracellular glutamate and increased NMDA receptor-mediated calcium influx, thereby contributing to neuronal hyperexcitability and potentially inducing excitotoxicity and neuronal loss [27,31,87]. However, further studies are needed to determine the exact mechanism of neuronal death after FS.

In this study, we performed the first differential analysis of neuronal loss in the dorsal and ventral hippocampus in an FS model. This regional assessment revealed an important nuance: while neuronal loss in the dorsal hippocampus was confined to the CA1 subfield, more extensive damage was observed in the ventral hippocampus, with cell loss evident in both CA1 and CA3 regions. This finding is significant as it suggests that the ventral hippocampus, which is more strongly implicated in emotional regulation, may be particularly vulnerable to functional impairment early in life.

### 4.2. The Molecular Changes

Our findings demonstrate that neonatal FS induce long-term, region-specific alterations in the expression of iGluR and mGluR, as well as EAATs, with the most pronounced changes observed in the ventral hippocampus and medial prefrontal cortex. Earlier research provided fragmented insights, describing alterations in specific components like mGluR5, EAAT2, and GluA1 in the cortex of young animals [36,43] or reporting no changes in hippocampal NMDA subunits at similar time points [44]. Our systematic approach, in contrast, uncovers a broader and persistent dysregulation across multiple glutamatergic genes, revealing a complex, region-specific molecular footprint of early-life FS that underpins the diverse functional impairments.

Notably, in the ventral hippocampus and medial prefrontal cortex, FS led to reduced expression of *Grin2a* (ventral hippocampus only) and *Grin2b* genes, with a parallel downregulation of the obligatory *Grin1* subunit [88]. This coordinated downregulation points toward a general reduction in NMDA receptor density in these regions.

Discrepancies with some earlier reports, which found no changes in NMDA subunit gene expression [44,89], may be explained by differences in experimental design or the timing of assessment. Notably, those studies that did detect alterations often found them at the post-translational level, such as impaired GluN2A phosphorylation after repetitive FS [44] or sustained increases in GluN2B phosphorylation following prolonged seizures [89], highlighting that functional deficits can occur independently of transcriptional changes.

In the dorsal hippocampus of experimental rats, we found no changes in the ex-pression of the studied NMDA receptor subunits. *Grin2b* gene expression tended to decrease, but the differences did not reach statistical significance. However, we observed an elevated *Grin2a/Grin2b* gene expression ratio in the dorsal hippocampus of experimental rats. Assessing this ratio is critical for understanding NMDA receptor functionality, as the balance between the GluN2A and GluN2B subunits directly influences receptor kinetics, synaptic plasticity, and descending signaling [90]. The normal neurodevelopmental transition from predominant *Grin2b* expression prenatally to elevated *Grin2a* expression postnatally is required for the maturation of hippocampal synaptic networks. It has been suggested that decreased *Grin2a/Grin2b* ratio may lower the seizure threshold by enhancing NMDA receptor-mediated excitability [91]. Therefore, the increase in the *Grin2a/Grin2b* ratio observed in our model may represent a compensatory mechanism aimed at reducing the hyperexcitability of hippocampal neurons.

We found reduced mRNA levels of the AMPA receptor subunits *Gria1* and *Gria2* specifically in the ventral hippocampus and medial prefrontal cortex. This contrasts with earlier reports of increased GluA1 protein at acute time points [43], suggesting a dynamic temporal regulation of AMPA receptor expression post-FS. Our findings align with a delayed decrease in GluA2 protein reported in a different FS model [92]. The downregulation of *Gria1*, a key subunit for LTP induction [93], likely contributes to the synaptic plasticity and memory deficits we and others have observed [7]. Moreover, the reduction in *Gria2* implies an increased formation of Ca^2+^-permeable AMPA receptors, which can exacerbate neuronal Ca^2+^ loading and promote excitotoxic damage [94,95].

Beyond iGluRs, we extended our analysis to mGluRs, providing the first comprehensive profile of their transcriptional changes in this model. Transcript levels of group I mGluRs (*Grm1* and *Grm5*) were diminished in the ventral hippocampus and medial prefrontal cortex. These postsynaptic receptors normally enhance intracellular Ca^2+^ signaling and tune synaptic strength [96,97]. Their downregulation therefore likely exacerbates the impairment of glutamatergic synaptic function. The apparent discrepancy with a prior study showing increased *Grm5* expression probably reflects distinct temporal dynamics, as that analysis was performed at a much earlier post-FS interval.

We also identified significant alterations in the less-explored groups II and III mGluRs. Specifically, expression of *Grm3* and *Grm4* (mPFC), *Grm7* (ventral hippocampus), and *Grm8* (dorsal hippocampus) was reduced. These presynaptic receptors function as autoreceptors, suppressing glutamate release upon activation [96,98]. Their downregulation could thus impair this auto-inhibitory feedback, potentially leading to enhanced synaptic glutamate levels. Conversely, the elevated expression of *Grm7* and *Grm8* in the temporal cortex might reflect a region-specific, homeostatic adaptation to counter neuronal hyperexcitability.

Our analysis of glutamate transporters revealed a specific reduction in the expression of *Slc1a3* (EAAT1) and *Slc1a1* (EAAT3) within the ventral hippocampus, while *Slc1a2* (EAAT2) remained unchanged. Although EAAT2 handles the bulk of hippocampal glutamate clearance, the contribution of EAAT3 at specific synapses can be substantial (~20%) [99,100,101]. Critically, both glial EAAT1 and neuronal EAAT3 play non-redundant roles in fine-tuning extracellular glutamate dynamics [101,102,103]. Thus, their coordinated downregulation likely disrupts precise glutamate homeostasis in the ventral hippocampus, creating a permissive environment for excitotoxicity and network dysfunction, as demonstrated in knockdown models [101].

Thus, the induction of FS during a critical period of glutamatergic system development leads to long-term alterations in the expression of various glutamatergic system components. These alterations, in turn, may underlie the impairments in synaptic plasticity and behavior.

### 4.3. Linking Molecular Disruptions to Behavioral Deficits

Cognitive deficits represent one of the most consistently reported long-term consequences of early-life FS in animal models. A substantial body of evidence, using paradigms ranging from the Morris water maze and active avoidance to fear conditioning, has documented persistent memory impairments in both young [10] and adult animals [6,7,8,9] with a history of FS. In the present study, we employed a complementary set of behavioral assays—open field habituation, novel object recognition, and Y-maze spontaneous alternation—to probe different aspects of learning and memory that are particularly dependent on hippocampal and prefrontal function.

The observed habituation deficit in the open field aligns closely with our molecular and histopathological findings. This form of non-associative memory is known to require intact NMDA receptor signaling (involving *Grin1* and *Grin2a* [104] and is highly sensitive to ventral hippocampal dysfunction [105]—precisely the circuits where we documented the most severe neuronal loss and glutamatergic downregulation.

However, it cannot be ruled out that the poorer performance of experimental rats in the Open Field habituation test may be associated not with memory impairment per se, but with a deficit in exploratory behavior. We demonstrated that experimental rats were less active when exploring a novel environment (exhibiting reduced motor activity in the Open Field test). This lower baseline level of exploratory behavior in the experimental group may have influenced the results of the memory test [106].

Similar deficits in exploratory behavior were observed during the learning stage of the Novel Object Recognition test, when two identical objects are initially presented. Rats with a history of FS spent less time exploring the objects compared to control rats.

The reduced level of exploratory activity in rats that experienced FS in the neonatal period may be linked to the decreased expression of NMDA receptor subunit genes in the ventral hippocampus that we identified, since it has previously been shown that NMDA receptor blockade in the ventral hippocampus reduces exploratory behavior in the Open Field [107].

The prefrontal cortex, along with the hippocampus, plays a key role in the implementation of cognitive functions. Having extensive connections with various brain structures, including direct projections to the ventral hippocampus, it integrates emotional and cognitive components of behavior, which is necessary for working memory processes [21,22,23]. The prefrontal cortex-hippocampus circuit also is crucial for recognition memory in rodents [108].

There is compelling evidence from rodent studies that both NMDA and AMPA receptors in the frontal cortex are critically involved in working and recognition memory mechanisms [109,110,111]. In particular, multiple pharmacological investigations have demonstrated that when NMDA receptors (particularly GluN2B-containing) and AMPA receptors are blocked in the medial prefrontal cortex, rodents exhibit significant impairments in working memory performance [110].

A notable finding of our study was the absence of significant deficits in novel object recognition or Y-maze working memory, despite the substantial downregulation of key AMPA and NMDA receptor genes in the medial prefrontal cortex and hippocampus. This result aligns with the report by Mesquita et al., who also found working memory to be intact in adult rats after neonatal FS [112].

This apparent dissociation between molecular and behavioral findings can be interpreted in several ways. First, the medial prefrontal cortex is functionally heterogeneous. The observed molecular deficits might be more pronounced in subregions like the infralimbic and prelimbic cortices [113,114,115], which are critically involved in emotional regulation and extinction learning—functions that were impaired in our rats—rather than in the spatial and recognition memory circuits probed by our tests. Second, compensatory mechanisms involving other neurotransmitter systems (e.g., dopamine, acetylcholine) not assessed in this study might have preserved performance in simpler memory tasks. Finally, the Y-maze and standard novel object recognition test may lack the sensitivity to detect subtle deficits, requiring more challenging cognitive paradigms to unmask functional impairments.

That molecular changes do lead to functional behavioral consequences is clearly demonstrated in the field of anxiety regulation. Our data reveal a consistent hyperanxious phenotype, evidenced by reduced center time in the open field and increased self-grooming in the social test. This behavioral profile co-occurs with the most severe histopathological and molecular deficits in the ventral hippocampus, a key node in anxiety circuits [116]. Interestingly, this effect was test-specific, as anxiety-like behavior in the elevated plus maze was unaltered.

Differences in anxiety-related behavior observed across tests may be due to differences in the stress response of experimental and control animals. Rats typically experience higher levels of stress in the Open Field and Social Interaction tests compared to the Elevated Plus Maze, where the closed arms provide a safe haven. It is well known that various adverse environmental factors, including those that trigger febrile seizures, can have long-term effects on the development of stress-response systems [117]. However, further research is needed to fully understand this issue.

Reports on anxiety-like behaviors following neonatal FS in rodents remain inconsistent. While some studies, like ours, describe an increase in anxiety-like behavior [11,112], others have reported decreased anxiety [8,118]. Some authors have also reported the development of depressive-like changes in rats that experienced FS at an early age [11,12], but we have not studied these forms of behavior.

We also found no impairment of social behavior in rats that had undergone FS at an early age. These results support clinical studies that found no impairment of social functioning in adolescents with a history of FS [119].

In summary, the behavioral profile observed—characterized by heightened anxiety, reduced exploration, and impaired habituation, but spared working memory and social function—exhibits a compelling regional alignment with the most pronounced molecular and cellular deficits in the ventral hippocampus and medial prefrontal cortex. This selectivity underscores the vulnerability of specific limbic circuits to early-life FS and provides a neurobiological framework for understanding the ensuing neuropsychiatric sequelae.

### 4.4. Conclusion and Future Directions

Our study demonstrates that neonatal FS lead to long-term, region-specific alterations characterized by neuronal loss in the hippocampus and a persistent downregulation of glutamatergic signaling components—including ionotropic, metabotropic receptors, and excitatory amino acid transporters—primarily within the ventral hippocampus and medial prefrontal cortex. These molecular and cellular deficits are accompanied by a distinct behavioral phenotype of increased anxiety, reduced exploratory drive, and impaired habituation, while working memory and social interaction remain intact.

While our study delineates robust correlations, establishing causality requires further investigation. Our previous electrophysiological work in the same model demonstrated impaired hippocampal LTP that was rescued by D-serine, an NMDA receptor co-agonist [7]. The NMDA receptor hypofunction suggested by our current transcriptional data, combined with this pharmacological evidence, positions D-serine supplementation as a compelling therapeutic strategy worthy of future behavioral testing in this model, consistent with its promise in other neuropsychiatric conditions [120,121,122,123].

Furthermore, the distinct alterations in mGluR gene expression provide a strong rationale for exploring their specific contributions to the FS phenotype. Given the established role of mGluRs in modulating synaptic transmission and plasticity, and the availability of selective pharmacological tools [124,125,126], future studies should employ these ligands to directly test whether correcting mGluR dysfunction can ameliorate the behavioral deficits we observed.

However, this study has several limitations that highlight the need for further research. First, the analysis was conducted at a single time point (P45-55). Tracking the age-dependent dynamics of these changes—from the acute post-seizure period through adolescence into adulthood—would reveal the developmental trajectory of the observed deficits and identify potential critical windows for intervention. Second, the reported mRNA levels should be interpreted as a proxy for transcriptional activity and may not directly correlate with the final protein abundance or functional activity of the receptors due to potential post-transcriptional and post-translational regulation. Confirming these findings at the protein level and using electrophysiological techniques is essential to determine whether the observed transcriptional changes translate into functional impairments in synaptic transmission and plasticity.

Furthermore, the present study was conducted exclusively in male rats. Given known sex differences in neural development and seizure susceptibility [8,127], a direct comparison of the long-term consequences of FS in males and females is a crucial next step to fully understand the neurobiological impact of early-life seizures.

It is important to note the limitations of our rat model when generalizing the conclusions to human anxiety risk. The model does not account for the full spectrum of human genetic vulnerability and environmental influences that shape clinical anxiety. The anxiety-like behaviors we measured, while translationally relevant, are not identical to the subjective experience and diagnostic criteria of human anxiety disorders. These limitations, however, highlight key directions for future research, such as validating these molecular findings in human post-mortem studies or using more complex genetic models to better mimic the human condition.

Furthermore, the hyperthermia model cannot fully replicate febrile seizures associated with infectious fever, as it lacks exposure to bacterial agents, which significantly increase the risk of severe neuroinflammation.

Collectively, our findings delineate a robust link between early-life FS, selective disruption of the glutamatergic system in limbic circuits, and specific long-term behavioral impairments. This precise identification of vulnerable circuits and molecular targets provides a foundation for developing targeted therapeutic strategies aimed at preventing or mitigating the neuropsychiatric consequences of FS.

## Figures and Tables

**Table 1:** Total number of animals in the study.

Group	Total Number	Behavioral Testing	mRNA Expression Analysis	Histology
Control	23	17	8	6
FS	17	12	8	5

## Data Availability

The data presented in this study are available on request from the corresponding author.

## Abbreviations

The following abbreviations are used in this manuscript:

AMPA	α-amino-3-hydroxy-5-methyl-4-isoxazolepropionic acid
ANOVA	Analysis of Variance
CA	Cornu Ammonis (followed by number: CA1, CA3)
CaMKII	Ca^2+^/calmodulin-dependent protein kinase II
cDNA	complementary DNA
Ctrl	Control
DI	Discrimination Index
DNA	Deoxyribonucleic acid
EAAT	Excitatory amino acid transporter (followed by number: EAAT1, EAAT2, EAAT3)
EEG	Electroencephalography
FS	Febrile Seizures
*Gria1/2*	Gene names for AMPA receptor subunits (GluA1/2 proteins)
*Grin1*	Gene name for obligatory NMDA receptor subunit (GluN1 protein)
*Grin2a/2b*	Gene names for NMDA receptor subunits (GluN2A/2B proteins)
*Grm1-8*	Gene names for metabotropic glutamate receptors (mGluR1-8 proteins)
iGluR	Ionotropic glutamate receptor
IL-1β	Interleukin-1 beta
LTP	Long-Term Potentiation
mGluR	Metabotropic glutamate receptor (Groups: I, II, III)
mPFC	medial Prefrontal Cortex
mRNA	Messenger RNA
NMDA	N-methyl-D-aspartate
*p*	Postnatal day (followed by number: P10, P50)
PBS	Phosphate-Buffered Saline
PFA	Paraformaldehyde
RT-qPCR	Reverse Transcription quantitative Polymerase Chain Reaction
*Slc1a1*	Gene name for EAAT3 (Solute carrier family 1 member 1)
*Slc1a2*	Gene name for EAAT2 (Solute carrier family 1 member 2)
*Slc1a3*	Gene name for EAAT1 (Solute carrier family 1 member 3)

## Appendix A

**Table A1:** Reference gene stability values within the dorsal hippocampus of rats obtained by four algorithms with the RefFinder online tool.

Dorsal Hippocampus										
	Delta CT		BestKeeper		NormFinder		GeNorm		Comprehensive Ranking	
Rank	Gene	Average of STDV	Gene	Std Dev	Gene	Stability Value	Gene	Stability Value	Gene	Geomean of Ranking Values
1	*Pgk1*	0.23	*Pgk1*	0.09	*Rpl13a*	0.131	*Pgk1*	0.090	*Pgk1*	1.19
2	*Rpl13a*	0.23	*Ywhaz*	0.14	*Pgk1*	0.134	*Ywhaz*	0.090	*Ywhaz*	2.06
3	*Ywhaz*	0.24	*Rpl13a*	0.16	*Ywhaz*	0.160	*Ppia*	0.198	*Rpl13a*	2.21
4	*Ppia*	0.26	*Ppia*	0.17	*Ppia*	0.193	*Rpl13a*	0.210	*Ppia*	3.94
5	*Actb*	0.29	*Actb*	0.17	*Actb*	0.224	*Actb*	0.235	*Actb*	4.73
6	*Hprt1*	0.31	*Hprt1*	0.25	*Hprt1*	0.256	*Hprt1*	0.260	*Hprt1*	6.00

**Table A2:** Reference gene stability values within the ventral hippocampus of rats obtained by four algorithms with the RefFinder online tool.

Ventral Hippocampus										
	Delta CT		BestKeeper		NormFinder		GeNorm		Comprehensive Ranking	
Rank	Gene	Average of STDV	Gene	Std Dev	Gene	Stability Value	Gene	Stability Value	Gene	Geomean of Ranking Values
1	*Ppia*	0.29	*Rpl13a*	0.22	*Ppia*	0.085	*Actb*	0.093	*Ppia*	1.68
2	*Gapdh*	0.30	*Ppia*	0.36	*Gapdh*	0.182	*Gapdh*	0.093	*Gapdh*	1.86
3	*Actb*	0.30	*Gapdh*	0.38	*Actb*	0.186	*Pgk1*	0.208	*Actb*	2.59
4	*Pgk1*	0.33	*Pgk1*	0.41	*Pgk1*	0.227	*Ppia*	0.231	*Pgk1*	3.72
5	*B2m*	0.41	*Actb*	0.43	*B2m*	0.334	*B2m*	0.294	*Rpl13a*	3.83
6	*Rpl13a*	0.45	*B2m*	0.44	*Rpl13a*	0.400	*Rpl13a*	0.347	*B2m*	5.23

**Table A3:** Reference gene stability values within the temporal cortex of rats obtained by four algorithms with the RefFinder online tool.

Temporal Cortex										
	Delta CT		BestKeeper		NormFinder		GeNorm		Comprehensive Ranking	
Rank	Gene	Average of STDV	Gene	Std Dev	Gene	Stability Value	Gene	Stability Value	Gene	Geomean of Ranking Values
1	*Gapdh*	0.19	*Actb*	0.14	*Gapdh*	0.102	*Actb*	0.069	*Gapdh*	1.19
2	*Actb*	0.20	*Gapdh*	0.18	*Actb*	0.124	*Gapdh*	0.069	*Actb*	1.41
3	*Hprt1*	0.22	*Rpl13a*	0.20	*Hprt1*	0.152	*Rpl13a*	0.158	*Rpl13a*	3.46
4	*Rpl13a*	0.23	*Pgk1*	0.23	*Rpl13a*	0.169	*Hprt1*	0.193	*Hprt1*	3.66
5	*Ppia*	0.24	*Hprt1*	0.23	*Ppia*	0.188	*Ppia*	0.206	*Ppia*	5.23
6	*Pgk1*	0.25	*Ppia*	0.26	*Pgk1*	0.206	*Pgk1*	0.221	*Pgk1*	5.42

**Table A4:** Reference gene stability values within the medial prefrontal cortex of rats obtained by four algorithms with the RefFinder online tool.

Medial Prefrontal Cortex										
	Delta CT		BestKeeper		NormFinder		GeNorm		Comprehensive Ranking	
Rank	Gene	Average of STDV	Gene	Std Dev	Gene	Stability Value	Gene	Stability Value	Gene	Geomean of Ranking Values
1	*Pgk1*	0.30	*Rpl13a*	0.20	*Pgk1*	0.070	*Pgk1*	0.155	*Pgk1*	1.32
2	*Ywhaz*	0.32	*Ppia*	0.33	*Ywhaz*	0.167	*Ywhaz*	0.155	*Ywhaz*	2.00
3	*Ppia*	0.35	*Pgk1*	0.34	*Ppia*	0.218	*Hprt1*	0.187	*Ppia*	2.91
4	*Hprt1*	0.36	*Ywhaz*	0.40	*Hprt1*	0.258	*Ppia*	0.238	*Rpl13a*	3.34
5	*Rpl13a*	0.42	*Hprt1*	0.46	*Rpl13a*	0.320	*Rpl13a*	0.298	*Hprt1*	3.94
6	*B2m*	0.55	*B2m*	0.46	*B2m*	0.501	*B2m*	0.381	*B2m*	6.00
